# Sensitivity analysis for reproducible candidate values of model parameters in signaling hub model

**DOI:** 10.1371/journal.pone.0211654

**Published:** 2019-02-12

**Authors:** Kentaro Inoue

**Affiliations:** Department of Computer Science and Systems Engineering, Faculty of Engineering, University of Miyazaki, Miyazaki, Japan; Kyushu Institute of Technology, JAPAN

## Abstract

Mathematical models for signaling pathways are helpful for understanding molecular mechanism in the pathways and predicting dynamic behavior of the signal activity. To analyze the robustness of such models, local sensitivity analysis has been implemented. However, such analysis primarily focuses on only a certain parameter set, even though diverse parameter sets that can recapitulate experiments may exist. In this study, we performed sensitivity analysis that investigates the features in a system considering the reproducible and multiple candidate values of the model parameters to experiments. The results showed that although different reproducible model parameter values have absolute differences with respect to sensitivity strengths, specific trends of some relative sensitivity strengths exist between reactions regardless of parameter values. It is suggested that (i) network structure considerably influences the relative sensitivity strength and (ii) one might be able to predict relative sensitivity strengths specified in the parameter sets employing only one of the reproducible parameter sets.

## Introduction

Mathematical models for signal transduction pathway can support the understanding of molecular mechanism in the pathway and predict the dynamic behavior of molecular activity [1–6]. To construct a complete mathematical model, we require information pertaining to the experimentally known pathway, time-course and dose response of molecular activity, and model parameters such as phosphorylation and binding rates in a system. However, some of this information, in particular, the model parameters, is difficult or impossible to obtain or measure experimentally. Therefore, we must estimate the model parameter values to recapitulate experiments in simulations [7–9].

**Fig 1:**
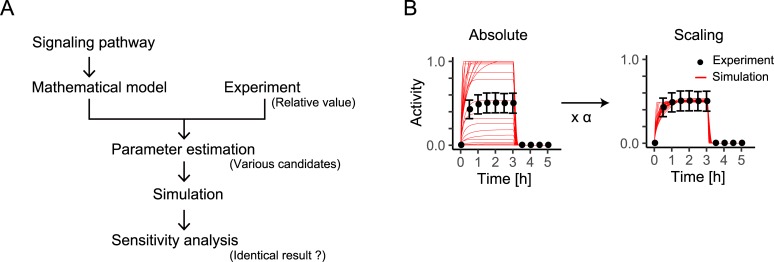
Overview of sensitivity analysis in signaling pathway model. (A) Overview of sensitivity analysis. (B) Values of signal activity measured experimentally are scaled in mathematical model.

Signal molecules in signal transduction pathway transmit extra-cellular information into transcription factors by activation, such as phosphorylation and ubiquitination. We can measure such activities but their values are relative abundances and not absolute abundances. A mathematical model must recapitulate the dynamic behaviors based on such experimentally relative abundances (**Fig 1**) [2, 3, 10]. However, some candidate parameter sets that can recapitulate the dynamic behavior of activities in experiments can be estimated because the combinations of the parameter values with the same dynamic behavior exist or the experimental data include noise and fluctuation.

To analyze the robustness of a model, sensitivity analysis has been implemented previously [11]. Local sensitivity analysis investigates an infinitesimal change in the target of a parameter set that can recapitulate experiments and can support features under a specific condition with known experiments. However, the sensitivity depends on the parameter values of the model. The common features for models with various reproducible candidates of model parameters are unclear.

In this study, we estimate diverse reproducible parameter values by parameter evaluation and analyze their characterization using local sensitivity analysis, focusing on the different and common features of sensitivity from reproducible parameter sets. The results show that although different reproducible model parameter values have absolute differences with respect to sensitivity strengths, specific trends of some relative sensitivity strengths exist between reactions regardless of parameter values. To the best of our knowledge, this is the first study to quantitatively investigate sensitivity and its relationships in reproducible parameter sets.

## Materials and methods

### Mathematical models and parameter estimation

**Fig 2:**
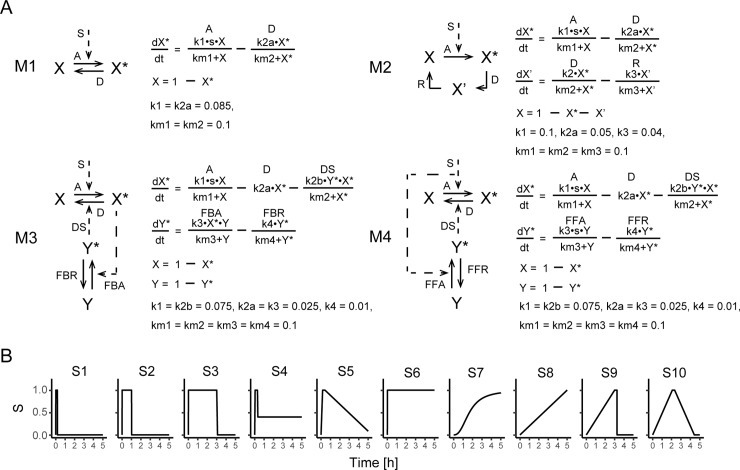
Network and mathematical model in signaling hub. (A) M1: Reversible reaction, M2: Cycle reaction, M3: Negative feedback loop, M4: Incoherent feedforward loop [12]. (B) Stimulation patterns of *s* in (A).

We used four models, as seen in the signaling pathway model (**Fig 2A**) [12]. These network structures resemble signaling hubs in well-known signaling pathways, such as p53, MAPK, or NF-κB pathway, and involve a reversible reaction (M1), a cycle (M2), a negative feedback loop (M3), and an incoherent feedforward loop (M4). The models are formulated considering Michaelis–Menten and mass action. These models have input signal patterns *s* of 10 different stimulations (**Fig 2B**). These input signal patterns express different combinations of “fast” and “slow” initiation and decay phases and can have specific respective effects on reactions in signaling hubs [12]. The functions and parameters of the input signal patterns are defined in **S1 Fig**. X* is the output.

First, we performed stochastic simulations using the Chemical Langevin equation (CLE) [13] with the original parameter values reported in Behar et al. [12] (**Fig 2A**), and generated activity value sets every 30 min as control data. For the stochastic simulation, the CLE was integrated using the Euler–Maruyama algorithm [14] that reproduces the discrete Wiener process.

$$dX(t)=\sum_{j=1}^{M}v_{j}a_{j}(X(t))dt+\sum_{j=1}^{M}v_{j}\sqrt{a_{j}(X(t))}dW_{j}(t),$$ (1)

where *v*_*j*_ indicates the stoichiometry, *M* is the number of reactions, *a*_j_(***X***(t)) is the propensity function for a reaction, and *W*_*j*_ (*t*) for 1≦*j*≦M are independent Wiener processes with gaussian noise *N*(0, 1).

To obtain the diverse parameter values in parameter estimation, the control data used were the stochastic simulation results in each parameter estimation round. Then, we used asynchronous genetic local search with distance independent diversity control (AGLSDC)—which combines local search with global search—[15] as the parameter estimation method, and estimated 1000 candidate sets for the control data. The fitness function was used as the cosine error [3] to obtain not the absolute values, but the dynamic behavior of the control data.

$$fitness=\sum_{i=1}^{N}\left(1-{\left(\frac{{\vec{x}}_{simulation,i}∙{\vec{x}}_{control,i}}{\left|{\vec{x}}_{simulation,i}||{\vec{x}}_{control,i}\right|}\right)}^{2}\right),$$ (2)

where *N* is the number of stimulation patterns, i.e., 10 in this case; ${\vec{x}}_{simulation,i}$ is a vector for values of X* in stimulation pattern *i*, obtained every 30 min for 300 min of the simulation; ${\vec{x}}_{control,i}$ is a vector for values of X* corresponding to the control data in stimulation pattern *i*; ${\vec{x}}_{simulation,i}∙{\vec{x}}_{control,i}$ is the inner product of ${\vec{x}}_{simulation,i}$ and ${\vec{x}}_{control,i}$, and |∙| denotes the magnitude of a vector. A low fitness means that the dynamic behaviors of the molecules in the simulation are similar to the ones in the control data. A scaling factor $\alpha =|{\hat{\vec{x}}}_{control,i}|/|{\vec{x}}_{simulation,i}|$, where $\left|{\hat{\vec{x}}}_{control,i}\right|$ is the mean of 1000 stochastic simulations, simulated with respect to the control data, can be calculated and scaled to the closest scale of the simulation to the control data (**Fig 1B**) [2, 3, 16].

In addition to the parameter estimation AGLSDC, we performed random sampling to collect 10000 parameter sets that could and could not recapitulate control data. Here, we defined positive data (reproducible parameter sets) as follows: a simulation produces positive data if it passes in standard errors from the averages ${\hat{\vec{x}}}_{control,i}$ every 30 min into the control data, which are calculated in the 1000 stochastic simulations. Otherwise, the data are negative. In this study, all the parameters were estimated in the range of log(-15) to log(5).

### Sensitivity analysis

The sensitivity of output X* to the *i*-th parameter was calculated as follows:

$$\frac{\partial lnq(p)}{\partial lnp_{i}}=\frac{\partial q(p)}{\partial p_{i}}\frac{p_{i}}{q(p)},$$ (3)

where *p*_*i*_ is the value of the *i*-th parameter, **p** is a vector *p*_*1*_, *p*_*2*_, …, *p*_*n*_, and q(**p**) is a target function. In this study, the target function used is a time-course integral of X*, which is a representative value of the dynamic behavior [17]. The sensitivity was numerically calculated with a 0.1% increase in the reaction rates.

### Implementation

We used the CVODE (http://computation.llnl.gov/casc/sundials/main.html) solver to perform numerical integration in the simulation. For the abovementioned analysis, the parameter sets involving errors in calculation by CVODE were excluded.

## Results

### Distribution of reproducible parameter sets in parameter space

**Fig 3:**
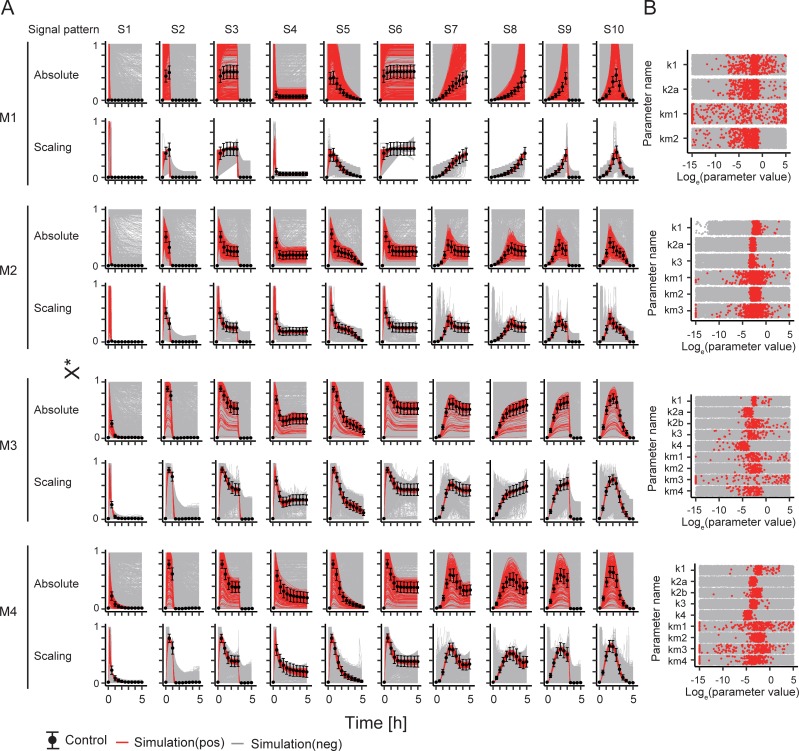
Absolute and scaling time-course dynamics and distribution of parameter values. (A) Time courses of absolute and scaling values in simulation. The gray and red lines indicate negative data and positive data, respectively. The black point and its error bar indicate the mean and standard deviation in stochastic simulation, respectively. (B) Distribution of parameter values.

We obtained positive data using AGLSDC and random sampling as follows: 546 in M1, 504 in M2, 112 in M3, and 169 in M4. These simulations can recapitulate dynamic behaviors but they are not necessarily consistent with the absolute values of the control data (**Fig 3A**). The estimated parameter sets were distributed in a wide range of values (**Fig 3B**). To confirm the details of the parameter values, we investigated the combinations of values of model parameters (**S2 Fig**). In M1, k1 and k2a in the positive data were correlated with a slight spread. The green circle of M1 in **S2 Fig** indicates partial correlation in high values of k1 and km1. These values supported the spread in k1 and k2a. The relationships between k1 and km1 were also seen in the other models (M2–M4 in **S2 Fig**). k3 in M2, M3, and M4 was also spread by km3. In M3, the parameter values between k2b and k3 or k4 were correlated. In M4, the parameter space of k2b was expanded by km2. These results indicate that the relationships among reproducible parameter values were correlated or partially correlated and balanced to recapitulate the control data. In particular, the rate parameters such as k1 and k2 exhibit correlation for reproducibility of dynamic behavior in control data, and the Michaelis constants can provide the expansion of parameter space. These results are consistent with those obtained when we carried out parameter estimation manually.

### Difference in distributions of sensitivity among signal patterns

**Fig 4:**
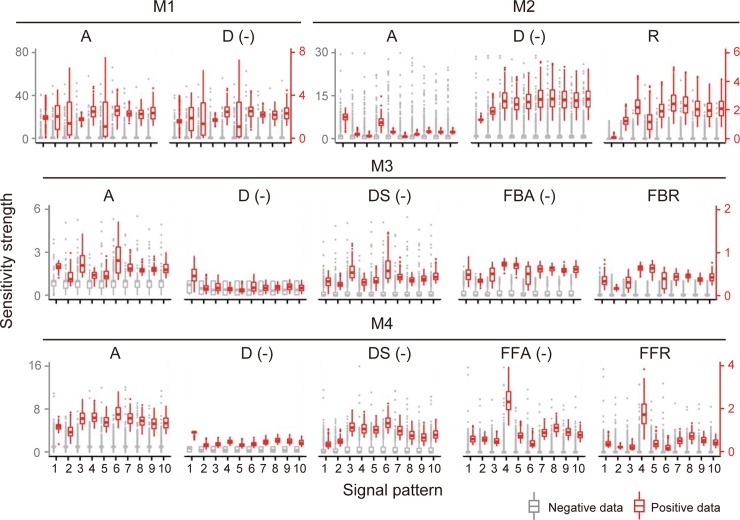
Distributions of sensitivity strength for time integral of X*. The gray and red lines indicate negative and positive data, respectively.

The section describes the results for the sensitivity analysis of the time-course integral of X* to positive and negative data and presents their distributions between signal patterns (**Fig 4**). In these models, the sensitivities for each reaction were clearly separated into positive or negative values in all the parameter sets. All the sensitivities of reactions D, DS, FBA, FFA exhibited negative values. This indicates that the increase in a reaction rate always causes increase or decrease in X* as per the specific reaction in these network structures.

In these models, the distributions of sensitivity in positive data were different between signal patterns (**Fig 4**). For example, the distributions of sensitivity of reactions A and D at signal pattern S4 in M1 were smaller than that at S6 in M1. These indicates that the range of sensitivity strength in positive data depends on the signal patterns. Furthermore, the distributions of sensitivity among rate parameters were different, which indicates that the influences of the rate parameters on X* were different. Overall, the distribution of sensitivity for reproducible parameter sets depends on the signal patterns and network structures.

### Statistical separation of sensitivity strengths between reactions at different signal patterns

**Fig 5:**
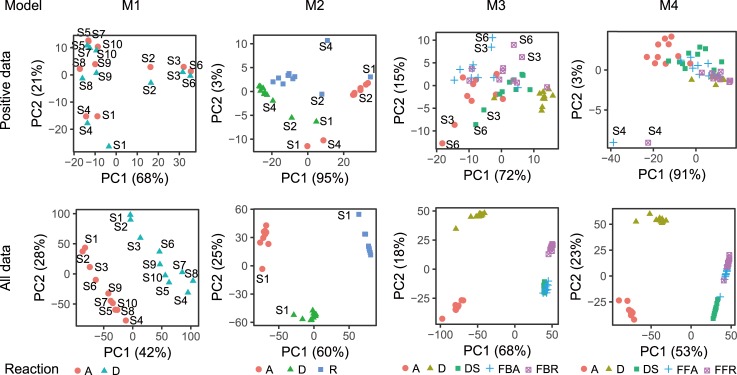
PCA of sensitivity strength in model. Shape indicates a reaction.

Next, we performed principal component analysis (PCA) for the sensitivity strength in each model to analyze the trends of sensitivity among reactions and signal patterns (**Fig 5**). In all the positive and negative data (bottom part of **Fig 5**), the principle components (PCs) of sensitivity were clearly separated into each reaction compared to those of the positive data (top part of **Fig 5**). The result indicates that the sensitivity strengths of each reaction for output X* were statistically different for all input signals, although the ratios of sensitivity strengths between reactions were different for different parameter sets (**S3 Fig**). In fact, the ranges of sensitivity strength in the negative data were different between reactions compared to differences between signal patterns (**Fig 4**). Besides, for the positive data, the PCs of sensitivity were different between input signal patterns except for the case of M1. For M1, the sensitivity strengths of reaction A and D at each signal pattern were similar (**Fig 5**); this is because the PCs were almost same between reactions at a given signal pattern (**S3 Fig**). In the other models, sensitivity strengths did not exhibit clear trends among reactions and signal patterns.

### Dependence of sensitivity strength on parameter values and its relative trends in a model

**Fig 6:**
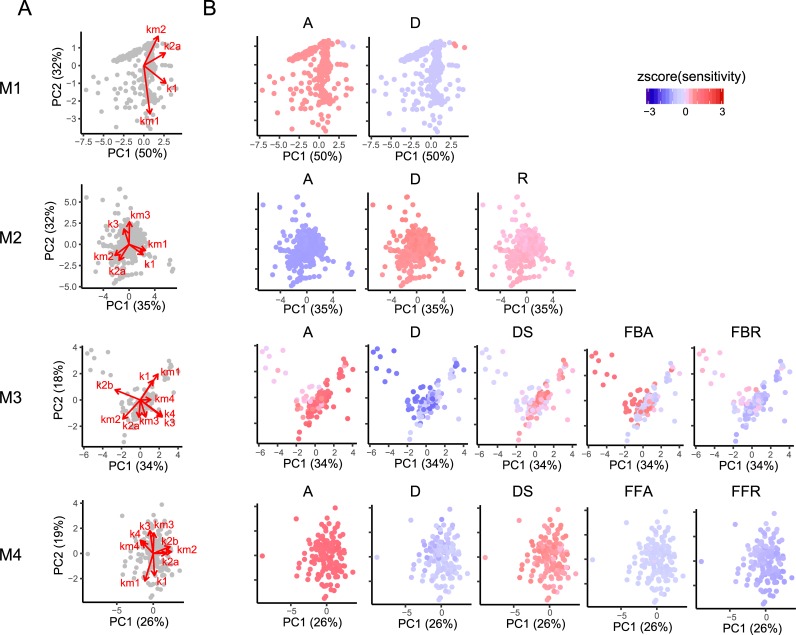
Relationship between model parameter and sensitivity at signal pattern S3. (A) Principal component loading and principal component of parameter sets in positive data. (B) Relative sensitivity strength of parameter sets at signal pattern S3. The color indicates the zscore of the sensitivity strength between reactions for each parameter set.

**Fig 7:**
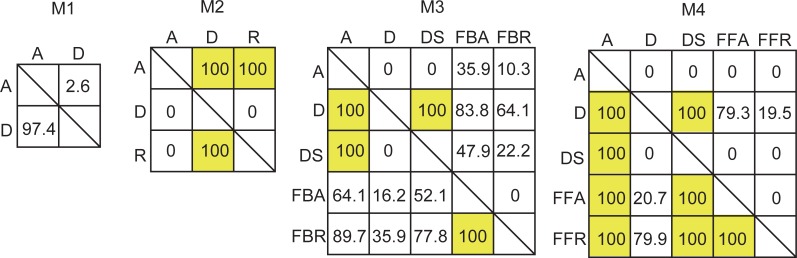
Qualitative comparison of reaction sensitivities at signal pattern S3. The presented values indicate percentages (%) at which the upper reaction has higher sensitivity than the left reaction in a given reproducible parameter set. For a given comparison, percentages not adding up to 100% indicate the existence of identical sensitivities, as exemplified by the cases of D and FFR in M4.

Next, we performed the PCA of parameter values to investigate the relative sensitivity strength of reactions for each parameter set (**Fig 6**). We present the heatmaps for the zscore of sensitivity strengths in each parameter set to compare the relative sensitivity strengths between reactions for a given signal pattern. The zscore is calculated from sensitivity strengths of all reactions at a signal pattern in a model for a parameter set to check whether the sensitivity for a given reaction is higher or lower than that for other reactions. At signal pattern S3 in M1, the sensitivity strengths for reaction A were higher than those for reaction D but the strengths for reaction D were higher when k2a was high. However, these strengths were nearly equivalent, as shown in **S3 Fig**. In M2, the relative sensitivity strengths between the reactions were same in any given parameter set. In M3, when k2b was high, the sensitivity of reaction FBA was higher, whereas in the other parameter sets, the sensitivity of reaction A was higher. In M4, when k3 and k4 were high, the sensitivity of reaction D was slightly lower. This indicates that the relative sensitivity strengths depend on a balance or combination of parameter values. To gain further insights, we qualitatively investigated percentages at which a given reaction has higher sensitivity than another reaction (**Fig 7**), showing that the sensitivities of some reactions in all models were always higher or lower than those of other specific reactions and concluding that these relative sensitivity strengths may be features of reproducible parameter sets. Overall, the results suggest that the relative sensitivity strengths between all reactions in a model are not necessarily the same for positive data and depend on the balance of parameter values and signal patterns (**Fig 6**, **S4 Fig**), whereas identical trends of the relative sensitivity strengths for specific reactions are obtained for all reproducible parameter sets (**Fig 7, S5 Fig**).

### Correlation between sensitivity and its target integral

**Fig 8:**
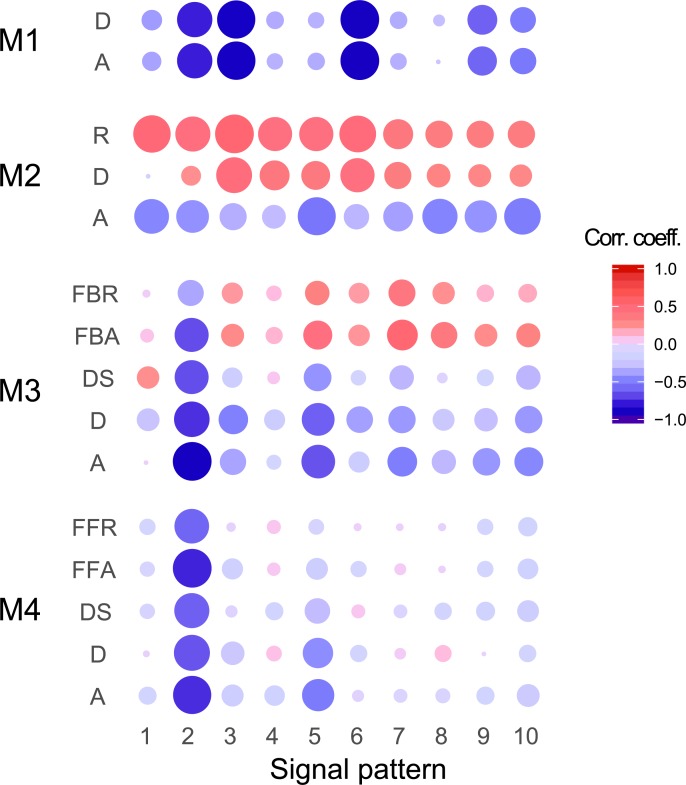
Correlation between sensitivity and its target integral. The size of circle indicates the value of the absolute correlation coefficient. Red and blue colors indicate positive and negative correlations, respectively.

Finally, we calculated the Pearson correlation coefficient between the integral and sensitivity to investigate the dependency of sensitivity target on the sensitivity strength (**Fig 8**). A negative correlation means higher sensitivity strength at lower integral and lower sensitivity strength at higher integral, while the opposite is true for positive correlation. The correlation coefficients at the signal pattern S2 and of reaction R in M2 were higher. In these reactions, the sensitivity strengths depend on the absolute integral without scaling (**Fig 3**). However, in most cases, the correlation coefficients exhibited low values. These results suggest that the influence of the absolute dynamic behavior on sensitivity is slight in most models.

## Discussion

We examined diverse parameter sets that can or cannot recapitulate experimental data to investigate the features of sensitivity in specific network structure and dynamics. We found that such reproducible parameter sets show different distributions and ranges of sensitivity strength between reactions and signal patterns. The relative trends of sensitivity strength in positive data were not necessarily the same between every reaction, but specific relative trends of sensitivity strength between specific reactions were always observed for reproducible parameter sets (**Figs 6 and 7 and S4 and S5 Figs**). These specific relationships of relative sensitivity strengths were found to be reliable sensitivity features for model prediction. Furthermore, these features imply that the prediction of relative sensitivity strengths specified in positive data may be accomplished using only one reproducible parameter set, because the trends of relative sensitivity strength are identical across reproducible parameter sets.

In previous work [18], the sensitivity from network topology in steady-state was analytically solved. It was shown that the network structure or topology determines the qualitatively positive or negative value of sensitivity. In the present study, all the values of sensitivity at each reaction also showed positive or negative trends (**Fig 4**). This may be approximately proved by the method given in a previous work [18]. Recently, quantitative or relative sensitivity strengths between reactions have become indicators for the target molecules of a disease [19]. Usually, the signaling system is important to the dynamic behavior after stimulation. When steady-state is assumed, we cannot know the dynamics of activity such as damped oscillations [3]. To understand the effect of reaction on such transient responses is an advantage of sensitivity analysis such as the numerical analysis performed herein. In our analysis, the absolute sensitivity strength was different for the reproducible parameter sets, but we could confirm that there are relative trends between sensitivity strengths in positive data.

In numerical analysis, the estimated parameter values depend on parameter estimation method employed. Therefore, we cannot know whether all parameter sets that recapitulate experimental data are accurately estimated. In this study, we checked the ranges and distributions of parameter values (**Fig 3B, S2 Fig**), which seem to be specific features of reproducible parameter values in positive data because negative data uses a wide range and shows region of non-reproducible parameter values. The correlation between model parameter values in positive data (**S2 Fig**) may be ascribed to the controlled balance of positive and negative regulation to fitness in parameter estimation. Differences between the trends of relative sensitivity strengths obtained for different parameter sets in positive data might be indicative of bifurcation, since simulations in positive data do not ensure exact control data (**Figs 6 and 7 and S4 and S5 Figs**). The results suggest that the positive data used in this study might be sufficient to determine the features of sensitivity in these models.

In this study, we focus on the local sensitivity analysis. However, global sensitivity analysis, which investigates change at a wide range of values in parameters, is also effective to understand the changes in dynamic behaviors for parameter change at a wide range of parameter values in a model [20]. We will investigate them in the near future. Another task to be considered in more detail is the parameter estimation. Current parameter estimation methods, such as genetic algorithms, find it difficult to estimate a large number of unknown parameter values in a large-scale and complex model. Thus, we used simple models in this study to evaluate the sensitivity features in estimable reproducible parameter sets. A better parameter estimation method is however required to understand a complex model.

## Supporting information

S1 Fig Equations (A) and parameters (B) used to define functions S1-10 in input signal patterns.
(PDF)

S2 Fig Distribution of parameter values in positive and negative data. The blue circle indicates correlation between parameters. The green circle and arrow respectively indicate partial correlation and its corresponding area to the spread of the parameter. Correlation or partial correlation between parameter values widens the parameter space in reproducible parameter sets.
(PDF)

S3 Fig Ratio of sensitivity between reactions. Ratio of sensitivity for each pair of reactions in a model. Log2(ratio) = 0 indicates that the sensitivity for the two reactions is equal. Gray denotes negative data, while red denotes positive data.
(PDF)

S4 Fig Sensitivity strength at PC for parameter values. PC1 and PC2 in (A) M1, (B) M2, (C) M3, and (D) M4 correspond to the ones in **Fig 6**. The color map shows the zscore of the sensitivity strength between reactions at each parameter set.
(PDF)

S5 Fig Qualitative comparison of sensitivity strength between reactions. The presented values indicate percentages (%) at which the upper reaction has higher sensitivity than the left reaction in a given reproducible parameter set. For a given comparison, percentages not adding up to 100% indicate the existence of identical sensitivities.
(PDF)
