# Supplementary material for: Sensitivity analysis for reproducible candidate values of model parameters in signaling hub model
Source: PLoS One. 2019 Feb 12;14(2):e0211654. doi: 10.1371/journal.pone.0211654 (PMC6372148; doi:10.1371/journal.pone.0211654)
Supplement: S1 Fig — Equations (A) and parameters (B) used to define functions S1-10 in input signal patterns. (PDF) [file pone.0211654.s001.pdf]

A

$$F_1(t) = \begin{cases} sb & t \leq td \\ sb + \dfrac{(tr - td)\, sh}{tr} & td \leq t \leq tr + td \\ sb + sh & tr + td \leq t \leq tp1 + tr + td \\ sb + sh - \dfrac{(t - tp1 - tr - td)\,(sh - sl)}{tc} & tp1 + tr + td \leq t \leq tp1 + tr + tc + td \\ sb + sl & tp1 + tr + tc + td < t \end{cases}$$

$$F_2(t) = \begin{cases} sb & t < td \\ \dfrac{sb(1+j) + (10^{\,t-td})^{h} - 1}{j + (10^{\,t-td})^{h}} & t \geq td \end{cases}$$

B

S	Function	sl	sb	sh	tr	td	tp1	tc	j	h
1	F1	0	0.005	1	0.5	0	10	0.5	NA	NA
2	F1	0	0.005	1	0.5	0	60	0.5	NA	NA
3	F1	0	0.005	1	0.5	0	180	5	NA	NA
4	F1	0	0.005	1	2.5	0	15	2.5	NA	NA
5	F1	0	0.005	1	10	0	15	300	NA	NA
6	F1	0	0.005	1	0.5	0	300	0.5	NA	NA
7	F2	0	0.005	1	NA	NA	NA	NA	100	2.5
8	F1	0	0.005	1	300	0	15	0.5	NA	NA
9	F1	0	0.005	1	180	0	15	0.5	NA	NA
10	F1	0	0.005	1	125	0	15	125	NA	NA

S1 Fig. Equations (A) and parameters (B) used to define functions S1-10 in input signal patterns.
