# Supplementary material for: Sensitivity analysis for reproducible candidate values of model parameters in signaling hub model
Source: PLoS One. 2019 Feb 12;14(2):e0211654. doi: 10.1371/journal.pone.0211654 (PMC6372148; doi:10.1371/journal.pone.0211654)
Supplement: S2 Fig — The blue circle indicates correlation between parameters. The green circle and arrow respectively indicate partial correlation and its corresponding area to the spread of the parameter. Correlation or partial correlation between parameter values widens the parameter space in reproducible parameter sets. (PDF) [file pone.0211654.s002.pdf]

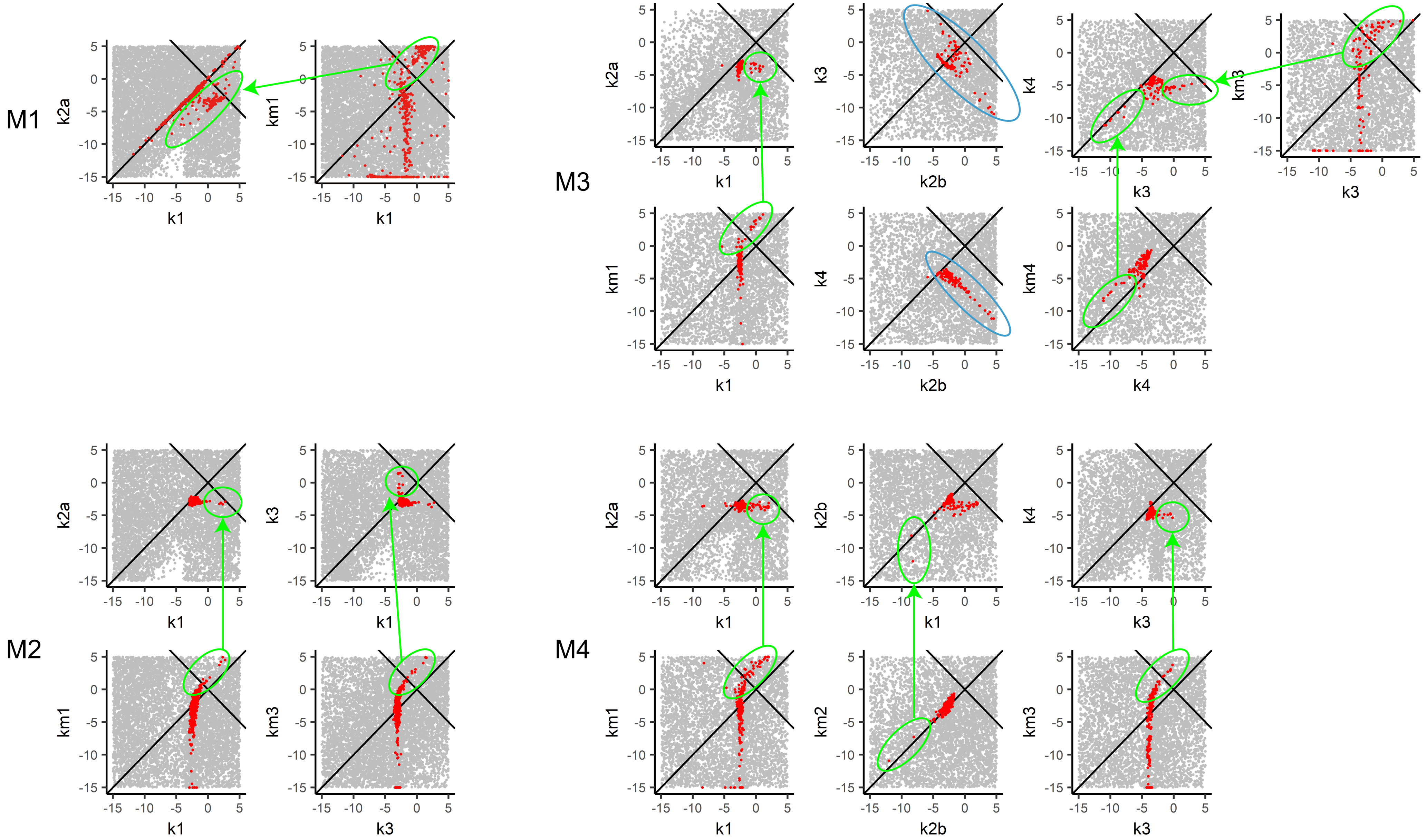

S2 Fig. Distribution of parameter values in positive and negative data.
The blue circle indicates correlation between parameters. The green circle and arrow respectively indicate partial correlation and its corresponding area to the spread of the parameter. Correlation or partial correlation between parameter values widens the parameter space in reproducible parameter sets.
