# Supplementary material for: Sensitivity analysis for reproducible candidate values of model parameters in signaling hub model
Source: PLoS One. 2019 Feb 12;14(2):e0211654. doi: 10.1371/journal.pone.0211654 (PMC6372148; doi:10.1371/journal.pone.0211654)
Supplement: S3 Fig — Ratio of sensitivity for each pair of reactions in a model. Log2(ratio) = 0 indicates that the sensitivity for the two reactions is equal. Gray denotes negative data, while red denotes positive data. (PDF) [file pone.0211654.s003.pdf]

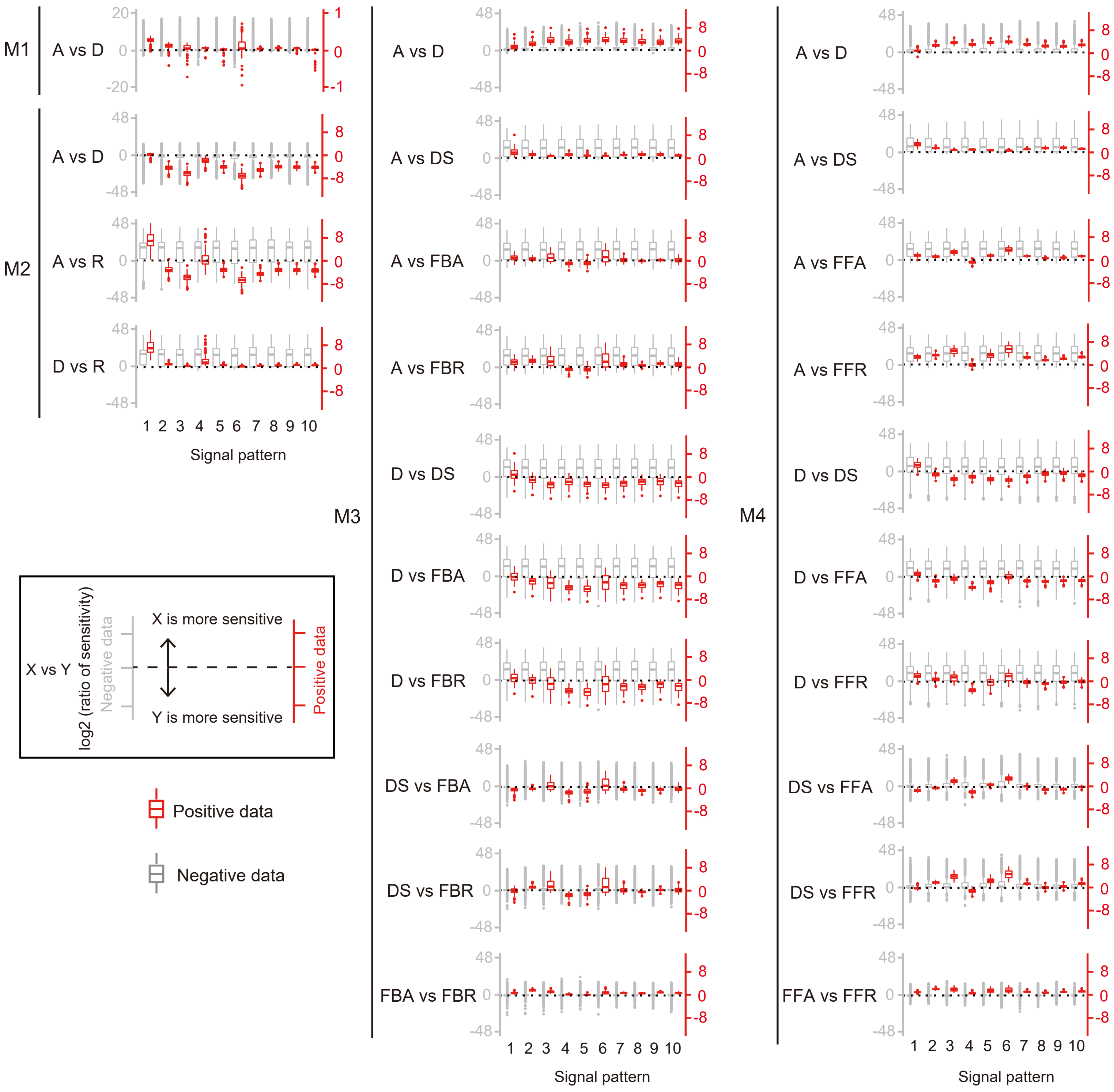

S3 Fig. Ratio of sensitivity between reactions.
Ratio of sensitivity for each pair of reactions in a model. Log2(ratio)=0 indicates that the sensitivity for the two reactions is equal. Gray denotes negative data, while red denotes positive data.
