# Supplementary material for: Sensitivity analysis for reproducible candidate values of model parameters in signaling hub model
Source: PLoS One. 2019 Feb 12;14(2):e0211654. doi: 10.1371/journal.pone.0211654 (PMC6372148; doi:10.1371/journal.pone.0211654)
Supplement: S4 Fig — PC1 and PC2 in (A) M1, (B) M2, (C) M3, and (D) M4 correspond to the ones in Fig 6. The color map shows the zscore of the sensitivity strength between reactions at each parameter set. (PDF) [file pone.0211654.s004.pdf]

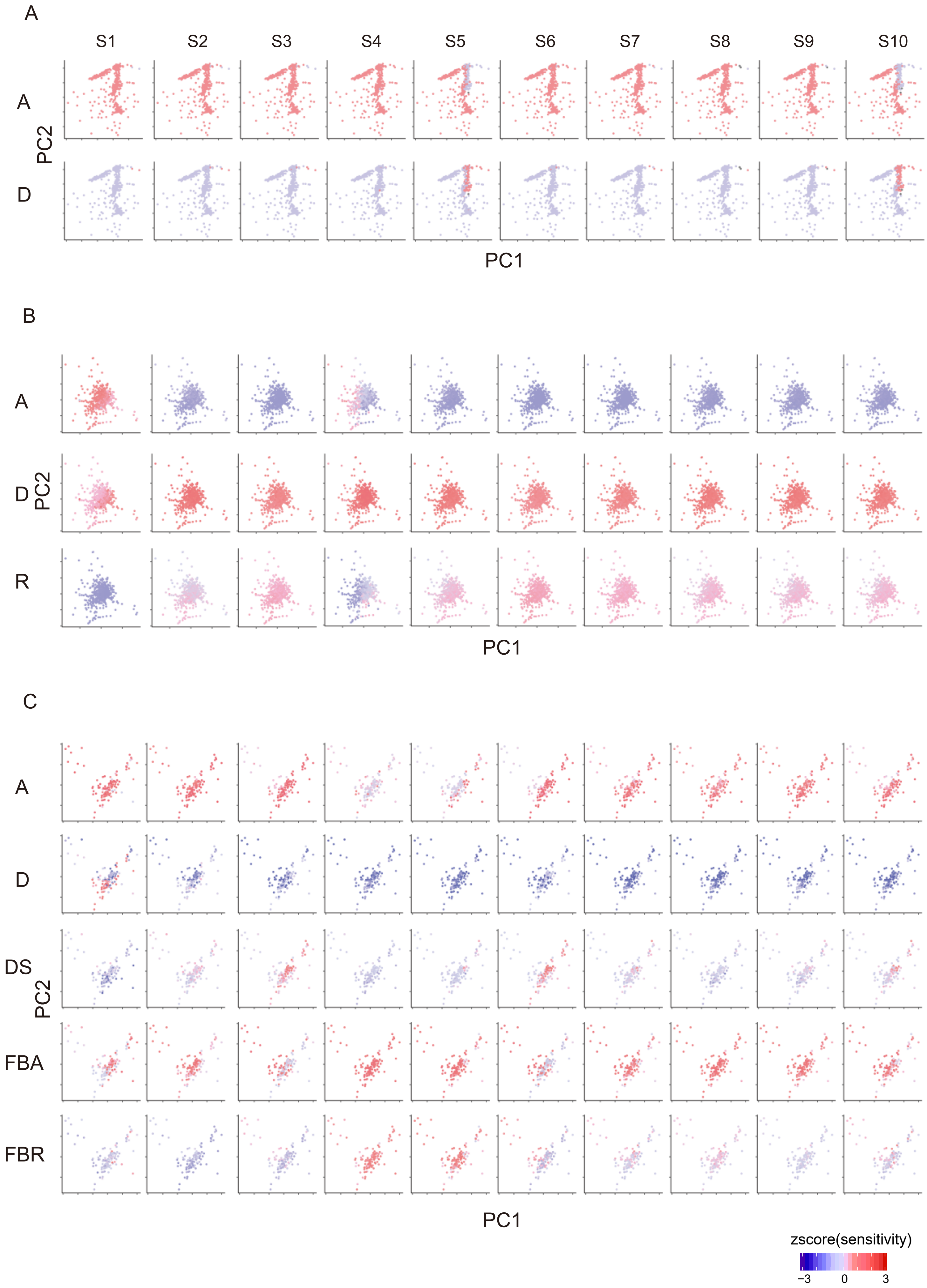

A
S1
S2
S3
S4
S5
S6
S7
S8
S9
S10
A
D
PC2
PC1
B
A
D
R
PC2
PC1
C
A
D
DS
FBA
FBR
PC2
PC1
zscore(sensitivity)
−3
0
3

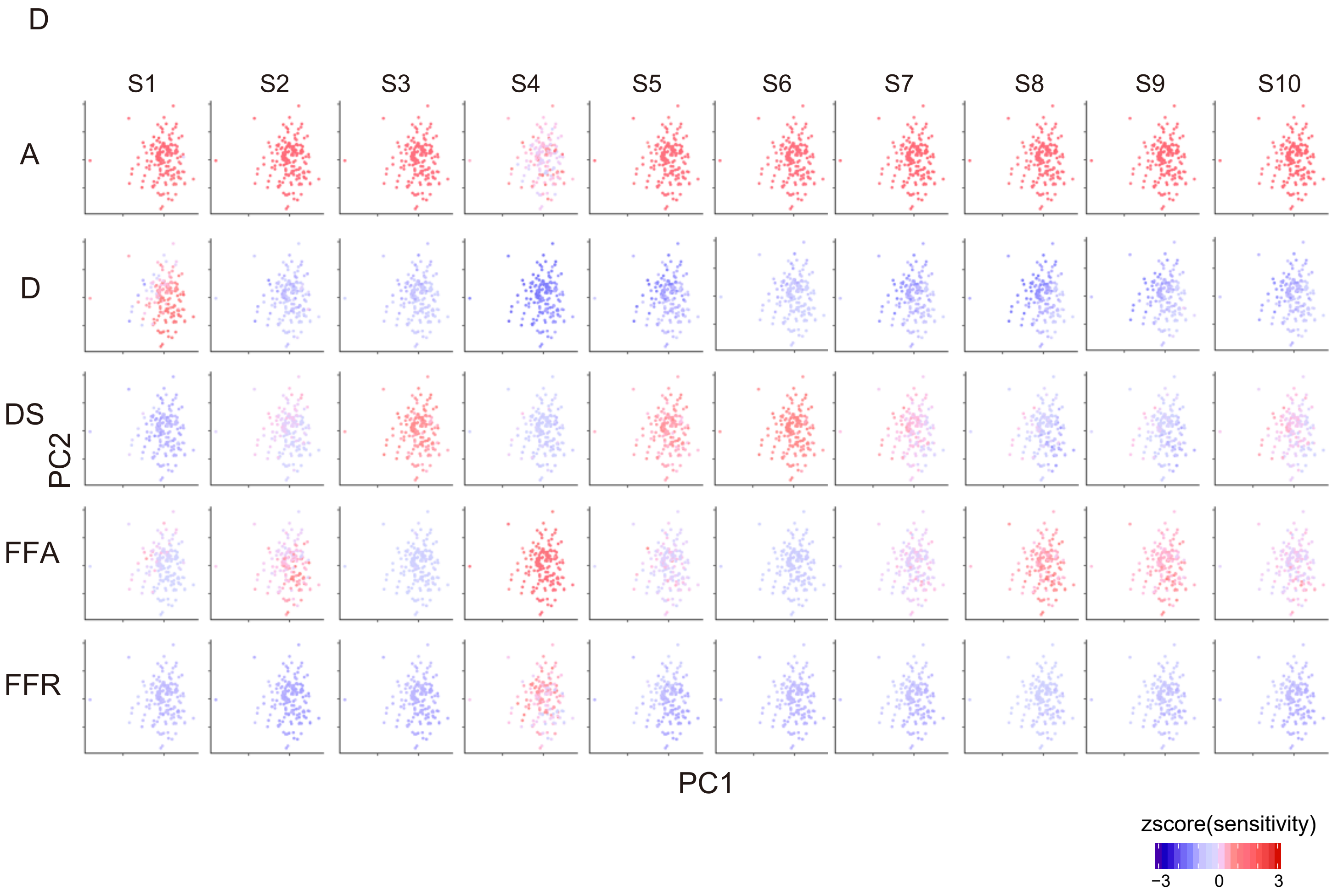

S4 Fig. Sensitivity strength at PC for parameter values.
PC1 and PC2 in (A) M1, (B) M2, (C) M3, and (D) M4 correspond to the ones in **Fig 6**. The color map shows the zscore of the sensitivity strength between reactions at each parameter set.
