# Supplementary material for: Sensitivity analysis for reproducible candidate values of model parameters in signaling hub model
Source: PLoS One. 2019 Feb 12;14(2):e0211654. doi: 10.1371/journal.pone.0211654 (PMC6372148; doi:10.1371/journal.pone.0211654)
Supplement: S5 Fig — The presented values indicate percentages (%) at which the upper reaction has higher sensitivity than the left reaction in a given reproducible parameter set. For a given comparison, percentages not adding up to 100% indicate the existence of identical sensitivities. (PDF) [file pone.0211654.s005.pdf]

A

S1

	A	D
A		0.4
D	99.6	

S2

	A	D
A		3.3
D	96.7	

S3

	A	D
A		2.6
D	97.4	

S4

	A	D
A		1.1
D	98.7	

S5

	A	D
A		29.3
D	70.5	

S6

	A	D
A		2.2
D	97.8	

S7

	A	D
A		0.5
D	99.5	

S8

	A	D
A		0.5
D	99.5	

S9

	A	D
A		0.2
D	99.5	

S10

	A	D
A		0.9
D	98.9	

B

S1

	A	D	R
A		25.7	0
D	74.3		0
R	100	100	

S2

	A	D	R
A		100	99.8
D	0		0
R	0.2	100	

S3

	A	D	R
A		100	100
D	0		0
R	0	100	

S4

	A	D	R
A		98.2	53.1
D	1.8		0
R	46.7	100	

S5

	A	D	R
A		100	100
D	0		0
R	0	100	

S6

	A	D	R
A		100	100
D	0		0
R	0	100	

S7

	A	D	R
A		100	100
D	0		0
R	0	100	

S8

	A	D	R
A		100	100
D	0		0
R	0	100	

S9

	A	D	R
A		100	100
D	0		0
R	0	100	

S10

	A	D	R
A		100	100
D	0		0
R	0	100	

C

S1

	A	D	DS	FBA	FBR
A		16.2	0.9	19.7	6.0
D	83.7		35.0	52.1	41.0
DS	99.1	65.0		100	54.7
FBA	80.3	47.9	0		0
FBR	94.0	59.0	45.3	100	

S2

A	D	DS	FBA	FBR
	0	0	23.1	0
100		74.4	94.0	43.6
100	25.6		91.5	0.9
76.1	6.0	8.5		0
100	56.4	99.1	100	

S3

A	D	DS	FBA	FBR
	0	0	35.9	10.3
100		100	83.8	64.1
100	0		47.9	22.2
64.1	16.2	52.1		0
89.7	35.9	77.8	100	

S4

A	D	DS	FBA	FBR
	0	0	98.3	88.9
100		92.3	100	100
100	7.7		100	100
1.7	0	0		0
11.1	0	0	100	

S5

A	D	DS	FBA	FBR
	0	0	80.3	74.4
100		99.1	100	100
100	0.9		94.0	91.5
19.7	0	6.0		6.8
25.6	0	8.5	93.2	

S6

A		0	0	36.8	24.8
D	100		100	71.8	61.5
DS	100	0		45.3	35.9
FBA	63.2	28.2	54.7		0
FBR	75.2	38.5	64.1	100	

S7

	0	0	46.2	8.5
100		99.1	100	96.6
100	0.9		85.5	54.7
53.8	0	14.5		0
91.5	3.4	44.4	100	

S8

	0	0	65.0	6.8
100		94.8	100	100
100	4.3		100	88.0
35.0	0	0		0
93.2	0	12.0	100	

S9

	0	0	38.5	0
100		91.5	100	94.9
100	8.5		98.3	40.2
61.5	0	1.7		0
100	5.1	59.8	100	

S10

	0	0	52.1	18.8
100		99.1	100	94.0
100	0.9		78.6	44.4
47.9	0	21.4		0
81.2	6.0	55.6	100	

D

S1

	A	D	DS	FFA	FFR
A		5.3	0	0.6	0
D	94.7		4.7	15.4	3.0
DS	100	95.3		100	70.4
FFA	99.4	84.6	0		0
FFR	100	97.0	29.6	100	

S2

A	D	DS	FFA	FFR
	0	0	0.6	0
100		91.7	98.2	14.2
100	8.3		81.1	0
99.4	1.8	18.9		0
100	85.8	100	100	

S3

A	D	DS	FFA	FFR
	0	0	0	0
100		100	79.3	19.5
100	0		0	0
100	20.7	100		0
100	79.9	100	100	

S4

A	D	DS	FFA	FFR
	0	0	95.9	59.8
100		100	100	100
100	0		100	91.1
4.1	0	0		0
39.6	0	8.9	100	

S5

A	D	DS	FFA	FFR
	0	0	0.6	0
100		100	100	55.0
100	0		4.1	0
99.4	0	95.9		0
100	45.0	100	100	

S6

A		0	0	0	0
D	100		100	53.3	21.3
DS	100	0		0	0
FFA	100	46.7	100		0
FFR	100	78.7	100	100	

S7

	0	0	0	0
100		100	100	60.9
100	0		27.8	0
100	0	72.2		0
100	39.1	100	100	

S8

	0	0	0	0
100		81.7	100	89.3
100	18.3		99.4	45.6
100	0	0.6		0
100	10.7	54.4	100	

S9

	0	0	0	0
100		76.3	99.4	61.5
100	23.7		99.4	21.3
100	0.6	0.6		0
100	38.5	78.7	100	

S10

	0	0	0	0
100		97.6	100	44.4
100	2.4		45.6	0
100	0	53.8		0
100	55.6	100	100	

S5 Fig. Qualitative comparison of sensitivity strength between reactions.
The presented values indicate percentages (%) at which the upper reaction has higher sensitivity than the left reaction in a given reproducible parameter set. For a given comparison, percentages not adding up to 100% indicate the existence of identical sensitivities.
